# With the Help of Kin? Household Composition and Reproduction in The Netherlands, 1842–1920

**DOI:** 10.1007/s12110-015-9222-y

**Published:** 2015-02-26

**Authors:** Paul P. P. Rotering, Hilde Bras

**Affiliations:** Department of Social Sciences, Wageningen University, PO Box 8130, 6700 EW Wageningen, The Netherlands

**Keywords:** Kin, Co-residence, Birth spacing, Inclusive fitness, The Netherlands

## Abstract

Relatives play an important role in human reproduction according to evolutionary theories of reproductive behavior, but previous empirical studies show large differences in the effects of kin on fertility outcomes. In our paper we examine the effect of co-resident kin and non-kin on the length of birth intervals over the reproductive life course of Dutch women born between 1842 and 1920. We estimate Cox proportional hazard models for parity progression based on the presence of kin and non-kin in the household while controlling for a large number of individual and community-level characteristics. We find that couples living with their brothers experienced shorter birth intervals whereas couples residing with a widowed father had relatively longer birth intervals. The effects of these types of kin on reproduction were most pronounced up to the birth of the fifth child, but not thereafter. We found no effect for mothers or other types of kin.

Co-resident kin and non-kin may play an important role in human reproduction (Johow and Voland 2012; Sear and Coall 2011; Tymicki 2004). Because of the relatively short interval between successive births and the long period during which newborns are dependent on others for their nutrition, parents behave as “cooperative breeders” (Hrdy 2005, 2008). This means that parents rely on other people—alloparents—who provide assistance in the form of care or resources, thereby helping to raise offspring and enabling parents to increase their reproductive outcomes (Hrdy 1999, 2009; Kramer 2010). According to Hamilton’s rule, kin assist in producing and raising offspring because of the indirect fitness benefit that this cooperative behavior yields (Hamilton 1964a, b). Recent empirical studies, many of which cover observations from contemporary and historical pre-transition societies, find that reproductive outcomes are indeed associated with the availability of kin assistance (Pollet et al. 2007; Voland and Beise 2002). Research suggests that among the most important caregivers are the couple’s parents (Hawkes et al. 1997, 1998; Tymicki 2004); their children, referred to as “helpers-at-the-nest” (Crognier et al. 2001; Kramer 2005); and the siblings of the couple (i.e., aunts and uncles of the newborn child) (Draper and Hames 2000; Feng et al. 2010; Hill and Hurtado 2009; Sear et al. 2003; Sear and Mace 2008).

However, empirical results vary because different categories of kin do not always influence fertility in the same manner (Sear and Coall 2011). These differential outcomes suggest that the effects of kin help on fertility may be contingent on specific local conditions and economic factors (Hames and Draper 2004) or that the effects of kin may vary over the life course of women as they progress from one birth to the next. Yet, few studies have systematically investigated the differential effects of kin on reproductive outcomes over the reproductive life course. Moreover, thus far only some studies have taken account of the influence of a wider group of kin and non-relatives (Bereczkei 1998; Lyngstad and Prskawetz 2010). The large amount of attention that has been given to parental influence has left the role of co-resident siblings, cousins, and non-kin underexplored (Nath et al. 2000; Snopkowski and Sear 2013; Voland and Beise 2002). One of the main reasons for the omission of the wider group of kin and other co-residents in many studies is the scarcity of sources that encompass detailed information on both the reproductive behavior of the couple and changes in the presence of kin and non-kin within the household over longer periods of time.

In this study, we exploit data on changes and differences in the composition of Dutch households from the second half of the nineteenth century until the beginning of the twentieth century in order to examine the effect of co-resident (non-)kin on the length of birth intervals over the reproductive life course. Focusing on sequential fertility outcomes over the life courses of the women in our sample allows for a more accurate investigation of kin effects than examining kin influences on total fertility or starting and stopping behavior. We use a rich data source, the Historical Sample of the Netherlands, which enables us to uncover the direction, magnitude, and significance of the effects of co-resident kin and non-kin on the reproductive careers of Dutch women born between 1842 and 1920. We develop our hypotheses on kin effects on fertility using insights from inclusive fitness theory.

Inclusive fitness theory is derived from evolutionary biology and concerns the natural selection of traits, such as altruism, which increase the genetic success of an organism (Hamilton 1964a, b; Hrdy 2008; Mace 2014). The main assumption of inclusive fitness theory is that humans—as do all species—strive to allocate their resources, including support, knowledge, and time, in such a way that they maximize their *inclusive fitness,* expressed as the number of kin weighted by the relative presence of one’s genes, or alleles (Hamilton 1964a, b; Hrdy 2008, 2009). In general, inclusive fitness theory thus suggests that people are driven to increase the fertility of their lineal and collateral kin in order to ensure the persistence of their genes in future generations. As long as the marginal benefits and costs of such assistance are in equilibrium, alloparental caregiving is likely to positively affect the number and survival chances of a person’s relatives and thus confers an indirect fitness benefit (Grafen 1984). It follows that if couples can rely on close kin members for support, they are more likely to raise more and/or better-quality children than couples who are not helped by kin (Kaptijn et al. 2010; Kramer 2010; Salmon and Shackelford 2008; Schaffnit and Sear 2014). Inclusive fitness theory has thus been invoked to help understand the relatively long post-generative life span of women. The “grandmother hypothesis” suggests that even though postmenopausal mothers no longer reproduce, they can still contribute to their inclusive fitness by providing resources or care to their children and grandchildren, thereby enabling them to increase their fertility or child survival rates (Hawkes 2003; Hawkes et al. 1997, 1998). The grandmother hypothesis has received strong empirical support (Sear and Coall 2011), although the pronatal effects of the couple’s parents may differ between paternal and maternal parents (cf. Euler and Weitzel 1996; Pollet et al. 2007; Sear et al. 2003; Strassmann and Garrard 2011; Voland and Beise 2002) or are conditional on the family’s social class (Johow and Voland 2012).

We extend our analysis of kin influence beyond the couple’s parents by also taking into account the effects of the presence of the couple’s siblings, other relatives, and household members who have no genetic relationship to the couple. Based on inclusive fitness theory, we depart from the broad hypothesis that all close kin will behave cooperatively and exert a positive influence on reproduction. We posit that the higher the degree of genetic relatedness, the more stimuli people have to increase their relatives’ fertility. Genetically close relatives (e.g., the couple’s parents or siblings) are expected to behave more cooperatively—leading to shorter birth intervals—than genetically more distant relatives (e.g., the couple’s aunts and uncles). In addition, uncertainty about the degree or nonexistence of genetic relatedness lowers the likelihood of cooperative behavior. The “confidence of paternity hypothesis” predicts that investments in grandchildren are lower if they are related through sons than through daughters (see Strassmann and Garrard 2011). Moreover, the incurred fitness benefit of cooperative behavior toward kin is possibly lower for fathers of the wife or husband, as there may be a component of uncertainty regarding the genetic bond with their offspring. For this reason, we hypothesize that mothers of the couple—in particular, maternal mothers—have a more pronounced positive effect on a woman’s fertility than fathers of the couple have (Sear and Mace 2008). Accordingly, living with both parents or having a widowed mother will be associated with shorter birth intervals compared with living with a widowed father. The couple’s siblings are hypothesized to have a positive effect on reproductive outcomes because of their genetic relation to the couple (Bereczkei 1998; Feng et al. 2010). Finally, household members with no genetic relation to the couple are, from an evolutionary perspective, less likely to affect reproductive outcomes because their fitness is not affected by this behavior.

Although the presence of kin is hypothesized to be associated with shorter birth intervals in general, the effects of kin on fertility may vary over the reproductive life span of the women in our sample. Kin effects are hypothesized to be stronger for the early parities than for later parities for three reasons. First, drawing on Lyngstad and Prskawetz (2010), the first, formative years of the family mark a unique transition in the life course of the young couple. Alloparental support in the early years could be important in compensating for inexperience among new parents, learning about the nutritional needs of newborns, or supplying the extra resources needed to feed an additional mouth. Second, local cultural norms concerning kinship and fertility may affect the likelihood of living together with kin before the couple is able to establish their own household. Skinner refers to this particular process as a “launching-pad family system” in which living with parents is common in the first few years after the couple is married, and the newly formed couple establishes their own independent household only after this initial co-resident phase (Skinner 1997). When the couple lives with the parents for longer periods of time, economic conditions or health concerns of the elderly parents, who are likely less able to provide support, may be the main motivation for kin co-residence (Pebley and Stupp 1987). Lastly, women who do not constrain their reproduction are not likely to be affected by the presence of others who are in a position to provide pronatal support. The intrinsic motivation or biologically heritable specific fecundity of these women is by itself enough to ensure short birth intervals while the presence of kin likely does not affect their fertility at all.

In the next section, we discuss our sample, measurements, and methods. Subsequently, we present the results of our event history analysis showing to what extent the presence of particular kin and non-kin in the household was related to the timing of subsequent births. In the final section, we discuss our findings in light of the recent literature, our hypotheses, and the data and methods used.

## Data, Measurements, and Methods

### Co-Residence with Kin in The Netherlands

**Fig. 1:**
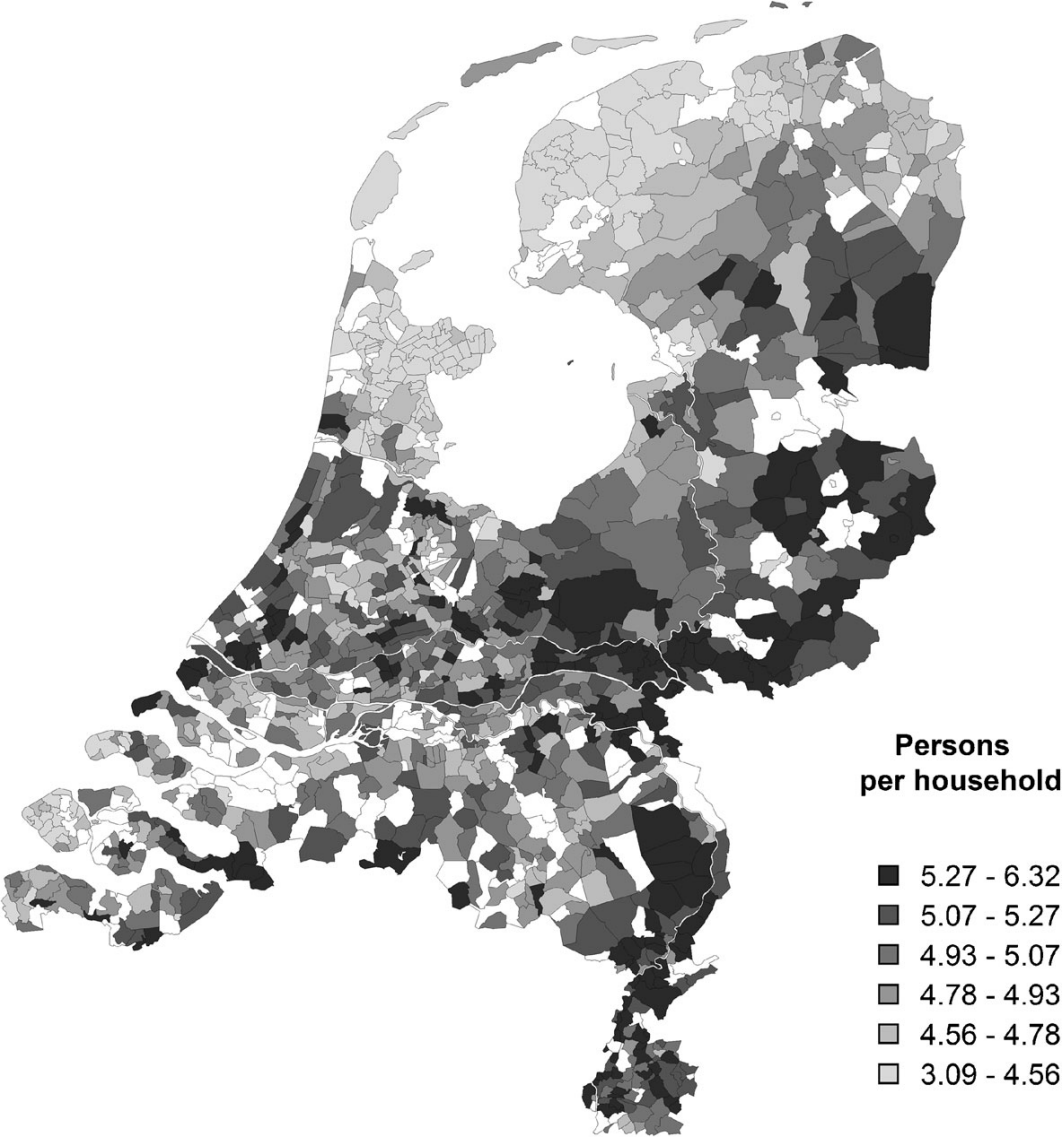
Number of persons per municipality, divided by the number of households per municipality. Data: Census 1899 (statline.cbs.nl). Cartography: NLGIS map of the Netherlands 1899 (Boonstra 2007)

The composition and size of nineteenth-century Dutch households varied considerably across regions and over time (Bras et al. 2010a; Kok and Mandemakers 2009; Kok et al. 2011). Kin co-residence in the Netherlands during the period of our analysis was primarily driven by altruistic motives to help kin, in particular those who were in need of help, and by rational motives, in particular in the eastern regions where co-residence was associated with inheritance practices (Kok and Mandemakers 2010). In her study on the dynamics of family structure in the textile town of Tilburg, Janssens (1993:84) concluded that poverty was not the sole reason for kin co-residence during the formative years of the family life cycle. In many cases, co-residence with immediate family occurred because it was the most practical option, for example, following a failed migration, the death of a parent, or because of the contributions kin could make to the household budget. The 1899 census indicates that 5.1 million Dutch individuals lived in about one million households, with household sizes ranging from 4.6 to 4.9 persons in the northwestern coastal provinces to 5.1 to 5.5 persons in the eastern provinces of Limburg, Gelderland, Overijssel, and Drenthe (Central Bureau of Statistics Statline, statline.cbs.nl). In the northwestern provinces, the nuclear family, or neolocal household formation, was the norm and most couples did not live together with their parents (Van der Woude 1970). Only in urban centers such as Amsterdam, Rotterdam, and Haarlem were households larger on average. The prevalence of co-resident kin was highest in the eastern provinces, and households in those regions were also much more likely to include persons who were not genetically related to the couple, such as boarders, servants, or lodgers (Kok and Mandemakers 2010). In terms of household size, the other provinces ranged somewhere in between (Fig. 1). Over time, co-residence with kin became less common in the Netherlands, in particular in the cities from the early twentieth century onward (Bras et al. 2010a).

### Data

The data used in the analysis were obtained from the Historical Sample of the Netherlands (HSN, release 2007, www.iisg.nl/hsn). The HSN is a representative, nationwide random sample of about 78,000 individuals (called “Research Persons”) born in the Netherlands between 1812 and 1922 (Mandemakers 2002, 2004). The main sources of the HSN are civil certificates and municipal population registers, which were established by royal decree on December 22, 1849. As of January 1, 1850, all municipalities began to keep population registers, based on the census of 1849, on a dynamic, continuous basis. Since 1861, all Dutch citizens are obligated to report events for recording in the population registers. Professional civil service workers were hired to maintain the registers. The main advantage of the HSN for our study is that people are followed from the cradle to the grave; individual life histories are not censored when individuals moved to another place in the Netherlands because their migration is recorded in the population registers. In addition, the date and place of birth or death, marital status, sex, religion, occupation, and relation to the head of the household are recorded for all members of the household. The exact date when a household member entered or left, due to birth, death, or migration, is known, including a reference to the place of origin or destination. From these registers, life courses have been reconstituted until 1939 when the registers were replaced by a system of personal cards. While the quality of the HSN is high and the observations in principle cover the entire country, there are some limitations to its use (Bras 2014; Knotter and Meijer 1995; Van Poppel et al. 2012; Vulsma 1988).^1^

First, maintenance of the population registers required all municipalities to continuously update the records, and when a person moved from one place to another, information had to be copied from other registers. This was not always done accurately (Knotter and Meijer 1995; Vulsma 1988). Second, since the nationwide registration of vital events started on January 1, 1850, the HSN does not include many complete life histories of women born before this date. Consequently, data from the 1860s and 1870s will mostly cover women born in the 1850s who had their first child at a relatively young age, causing a downward bias in the age at first birth for the earliest cohort. However, for the purpose of our analysis, this bias does not alter our conclusions on the effects of kin on the length of the interval between births. Third, the HSN is in continuous development, and data before 1883 has only been digitized for three provinces (Zeeland, Utrecht, and Friesland) and one city (Rotterdam). From 1883 on, the HSN has national coverage, and information is available for all 11 provinces. This gives the analytical sample we extracted from the HSN an urban bias and explains why the percentage of farmers and farmworkers (21.6%) is relatively small (Bras 2014; Van Poppel et al. 2012). Fourth, owing to the nature of the registers, our observations of kin are limited to those residing with the couple and exclude relatives living outside of, but in close vicinity to the couple’s house. This implies that the frequency of contact with others might have been higher in reality than is observed based on the presence of kin and non-kin in the household. Nevertheless, we assume that the presence of co-resident kin allows for frequent contact and is therefore a good and reliable measure of the availability of kin (Feng et al. 2010; Morgan and Rindfuss 1984; Schaffnit and Sear 2014; Snopkowski and Sear 2013; Tsay and Chu 2005).

For our analytical sample, we have selected couples (*N* = 2628) who entered a first marriage and for whom complete information is available about (1) the couple’s reproductive career until the woman is 50 years old and the husband is still alive and (2) the presence of kin and non-kin in the household. We focus only on the waiting time from the first until the second marital birth and on subsequent birth intervals but exclude the interval between marriage and first birth. The interval between marriage and first birth was often relatively short and in many cases not affected by kin presence, but instead by cultural norms which prescribed that the conception of the first child should follow not too long after marriage (Liefbroer and De Jong Gierveld 1995). Focusing on the interval between first and second or subsequent births reduces the possible influences of such cultural norms. Our analytical sample includes 2331 couples who had at least two children and 8052 closed birth intervals connecting to childbirths that took place between 1869 and 1939.

### Outcome Variable

Our outcome variable is the duration of the interval between first and subsequent live, marital births—i.e., parity two and up—measured in months. The birth interval is a useful indicator of fertility and is widely used in the literature on reproduction (e.g., Nath et al. 2000; Van Bavel 2004; Van Bavel and Kok 2004, 2010; Van Poppel et al. 2012). As in most nineteenth- and early-twentieth-century northwest European societies, fertility within marriage was generally regulated by prolonged breastfeeding, periodical or complete abstinence, *coitus interruptus,* and, to a lesser extent, abortion (Santow 1995). Birth spacing was part of the set of means to regulate fertility and was usually motivated by a desire to control family size. Life-cycle models of reproductive behavior indicate that variations in household income or expenditures resulting from the birth of children may produce an imbalanced ratio between “consumers” and “producers” in terms of household income or labor, resulting in economic stress and thereby necessitating fertility regulation (see, e.g., Alter 1988; Heckman and Walker 1990; Hotz et al. 1997). Underlying our hypotheses is the assumption that, from an evolutionary perspective, shorter birth intervals and an increase in child survival rates increase the fitness of women and the inclusive fitness of kin (Morgan and King 2001). However, reproduction is energetically costly to women and could jeopardize their health as well as that of their offspring, leading to a trade-off between quantity and quality of children (Borgerhoff Mulder 2000; Conde-Agudelo et al. 2006, 2007; Palloni and Millman 1986). Therefore, optimal birth intervals may be of intermediate length to ensure a sufficient number of offspring without exposing women and their offspring to excessive risk.

Table 1 reports the mean length of closed birth intervals by parity and for all births, as well as the total number of living children by birth cohort. Our sample covers the early period of fertility decline, between 1890 and 1920; the Dutch fertility transition was completed between 1920 and 1940 or even later in some regions (see Bras 2014). The average number of living children per woman declined over time, most strikingly since the 1881–1890 birth cohort. In line with Van Poppel et al. (2012), the mean length of birth intervals in our sample increased most significantly after the last quarter of the nineteenth century, from 26.4 months for women born before 1860 to 28.8 months for women born in 1901 or later. As Table 1 shows, this was in particular due to an increase in the length of the early parities, suggesting that spacing was increasingly used as a strategy to control childbirth, although stopping altogether (rather than delaying) was the main cause of Dutch fertility decline (Van Poppel et al. 2012). When we compare birth intervals over the life course, we observe that earlier cohorts (e.g., before 1860) experienced birth intervals that were increasing in length over the reproductive life span. In contrast, women born after 1900 had increasingly shorter birth intervals over their life courses and stopped having children sooner.

**Table 1:** Mean length of birth intervals in months by parity and mean total number of children by birth cohort of the wife

Birth cohort wife	Birth interval by parity (in months)						Total number of children^b^
	2	3	4	5	6 and up	Mean^a^	
All	27.8	28.9	28.5	27.5	25.7	27.6	4.56 (2.78)
<1860	25.1	26.1	27.6	25.8	27.2	26.4	5.71 (2.66)
1861–1870	24.9	26.7	26.9	26.4	26.0	26.1	5.14 (2.73)
1871–1880	23.8	26.9	27.2	27.0	24.0	25.5	5.34 (2.70)
1881–1890	26.4	29.0	27.6	27.8	26.0	27.1	5.31 (3.23)
1891–1900	29.8	30.4	31.2	29.7	26.3	29.4	4.20 (2.60)
>1900	29.7	30.3	28.7	26.0	21.6	28.8	2.93 (1.32)

^a^Excluding the interval between marriage and first birth

^b^Number of living children observed at last birth (standard deviation in parentheses)

Source: Historical Sample of the Netherlands, release 2007

### Independent Variables

We include the presence of one or more of the following kin and non-kin types as independent variables (all are relative to the couple): (1) parents; (2) siblings; (3) other relatives, such as cousins, aunts, and uncles; and (4) non-kin (servants, boarders, and lodgers). Since couples may live only part of their reproductive lives together with others, all kin and non-kin are coded as time-varying dummy variables with value 0 (not present) or 1 (at least one person of that particular kin type is present in the household of the couple). Table 2 provides descriptive information about the different variables used in the models, by parity. Co-residence with kin occurred frequently in the Netherlands, but during the life course of the couples in our sample, many relatives left the household. There were hardly any differences in the proportion of couples living with relatives of the husband or with relatives of the wife. Around a quarter of all couples lived with both parents of either the husband or the wife during the second parity (the interval between first and second birth). Over the life course, the share of couples living with both parents or with widowed parents of either spouse decreased to less than 10% and less than 5%, respectively. Co-residence with siblings was as common as living with parents. Here too, we observe no difference between relatives of the husband and relatives of the wife in terms of the share of households where these kin types lived, although co-residence with siblings of the wife occurred slightly more frequently in the highest parities (from the birth of the fifth child on). Co-residence with other types of kin (e.g., aunts or uncles of the couple) was less common and occurred in less than 5% of all cases for the first parity and decreased to around 1% for the highest parities. In our sample, co-residence with non-kin, such as servants or boarders, did not occur frequently. Servants were present in around 1 of all households and boarders were present in only a handful of cases. The share of households with non-kin did not vary significantly over the life course of the women in our sample.

**Table 2:** Descriptive statistics for variables used in the analysis, by parity

Variable	Parity (Percentages)^a^					*F* test^b^
	2	3	4	5	6 and up	
Household characteristics						
Kin present						
Wife, both parents	25.2	19.5	15.4	12.6	9.8	***
Wife, only father	9.8	9.4	7.1	6.8	4.1	***
Wife, only mother	6.5	6.8	6.1	5.8	4.5	*
Husband, both parents	24.5	20.1	16.6	13.1	8.5	***
Husband, only father	10.0	9.2	8.3	7.3	4.7	***
Husband, only mother	5.4	5.9	4.3	4.2	2.5	***
Wife’s sister(s)	26.1	20.4	17.6	15.1	10.5	***
Husband’s sister(s)	27.4	22.0	18.1	13.6	7.9	***
Wife’s brother(s)	27.1	22.5	18.0	16.0	11.9	***
Husband’s brother(s)	27.3	21.9	18.9	15.6	8.4	***
Other female kin	4.3	3.4	3.2	2.7	1.2	***
Other male kin	4.2	3.8	3.3	2.7	1.7	***
Servant(s)	1.0	1.0	1.0	1.2	1.1	
Boarder(s)	0.4	0.3	0.3	0.3	–	
Community characteristics						
Nuclear	60.4	59.0	57.4	57.0	53.4	
Stem	15.4	15.9	16.4	17.1	18.7	
Intermediate	24.2	25.1	26.2	26.0	27.9	
Urban	67.6	65.6	64.0	63.1	62.0	
Religion						
Both Roman Catholic	25.4	28.4	30.0	32.3	35.6	
Both Liberal Protestant	15.4	14.8	15.2	14.4	12.9	
Both Orthodox Protestant	31.3	31.1	30.6	29.4	28.0	
Mixed Catholic and Protestant	7.3	7.5	6.6	7.6	8.8	
Mixed Protestant	12.7	11.9	12.0	11.1	9.9	
Other or unknown religion	7.9	6.3	5.6	5.2	4.7	
Occupation of husband						
Higher manager	2.2	1.4	1.5	1.2	1.1	
Lower manager/cleric/salesperson	17.8	16.0	15.4	14.2	12.7	
Foremen or skilled worker	19.6	20.0	19.6	19.3	19.7	
Farmer or fisher	9.1	9.8	10.5	10.8	11.9	
Lower skilled worker	17.3	16.4	15.8	15.9	13.9	
Unskilled worker	15.1	16.5	16.4	16.4	15.4	
Lower or unskilled farmworker	12.5	13.5	14.6	15.1	17.4	
Unknown occupation	6.4	6.3	6.1	7.2	7.9	
Individual characteristics						
Mean age wife (in years)	26.1	27.8	29.5	31.1	34.6	
Mean duration of marriage (in years)	2.1	4.0	5.9	7.8	12.0	
Birth cohort wife						
<1860	6.4	8.7	10.6	11.4	10.2	
1861–1870	8.2	9.4	10.6	12.5	11.8	
1871–1880	10.2	12.3	14.4	15.1	15.3	
1881–1890	20.7	22.2	25.0	26.5	33.6	
1891–1900	26.7	25.3	24.0	23.2	23.1	
>1900	27.8	22.0	15.4	11.3	6.0	
Age distribution wife						
<25	42.7	27.4	14.4	5.9	0.5	
25–29	39.2	43.0	42.9	36.7	14.4	
30–34	15.0	23.6	32.0	38.9	36.8	
35–39	2.3	4.9	8.7	15.1	31.5	
>39	0.4	0.6	1.3	1.8	10.9	
Previous infant died						
Within 8 months	4.4	4.2	4.7	4.7	4.5	
After 8 months	0.8	1.6	1.7	2.0	2.4	
Age gap spouses						
Wife older	21.5	21.3	21.4	19.1	15.5	
Husband <6 years older	63.8	64.3	63.2	65.5	69.2	
Husband >6 years older	14.6	14.5	15.4	15.3	15.2	
Births (*N*)	2331	1727	1204	867	1923	

^a^Percentages rounded to the nearest tenth, unless otherwise stated

^b^Anova *F* test for difference between parity, significance thresholds: † *p* < 0.1, * *p* < 0.05, ** *p* < 0.01, *** *p* < 0.001

Source: Historical Sample of The Netherlands, release 2007

We control for several community-level characteristics. Based on population size and the percentage of the population working in agriculture, we indicate whether the household was located in a rural or urban setting. Owing to the overrepresentation of urban areas in the HSN, in particular for the earlier cohorts, more than half of all households are categorized as urban. Regional differences in social norms and attitudes toward kinship are captured by a categorical variable indicating the family system in the region of the household. Family systems are connected to the composition of households, the strength of kin ties, the inheritance of property, and norms and values regarding family relations and life course events (Bras et al. 2010a; Hilevych and Rotering 2013; Kok et al. 2011; Todd 1985). The northwestern coastal provinces, where partible inheritance was practiced and kin ties were relatively weak compared with other regions, are coded as *nuclear* family systems. The southeastern provinces are coded as *stem* family systems because of the occurrence of impartible inheritance, or *Anerbenrecht,* and the specific customs with regard to co-residence in which young couples “married in” and became part of the parental household. The remaining provinces are coded as *intermediate* family systems (Bras and Van Tilburg 2007).

At the individual level, we include the birth cohort of the wife to account for the general trend of fertility decline and increasing lengths of birth intervals over time, as discussed above. A woman’s age is one of the main determinants of fecundity and coital frequency, and thus connected to the duration of parity progression (Van Bavel and Kok 2004). In order to control for age effects, we include the age of the wife and the duration of marriage at childbirth. Both are expected to be associated with increasing birth intervals. Larger power differences between husband and wife have been linked to increased reproductive success (Bereczkei and Csanaky 1996; Voland and Engel 1990), and therefore we include categories for the age difference between husband and wife, coded as 0 (husband 0 to 5 years older), 1 (husband more than 5 years older), and 2 (wife older), as a crude proxy for power distance between spouses. We control in all models for the total number of children born, including deceased children. The premature death of the previous child may induce a replacement effect and thus may shorten the time to conception of the next child (Derosas 2006; Knodel 1982; Van Bavel and Kok 2004, 2010). In addition, since breastfeeding delays the return to ovulation, a child’s survival somewhat decreases a woman’s chance of becoming pregnant (Santow 1987). To control for these effects, we control for the death of the previous child within 8 months after birth or after 8 months since birth.

The HSN includes information on the religion of both husband and wife, which allows for coding all combinations of religious denominations as categorical dummy variables. The following categories are discerned, following Van Bavel and Kok (2004, 2005): liberal Protestants, orthodox Protestants, Catholics, “mixed,” and “other.” The first category, liberal Protestants, includes the majority of moderate and liberal schools in the Dutch Reformed Church and the liberal Protestant churches, such as Mennonites, Lutherans, and Remonstrants. When both spouses fall under this category, the couple is classified as liberal Protestant. The second category, orthodox Protestants, contains couples in which one or both spouses were members of the Calvinist church or belonged to the orthodox denomination in the Reformed Church. The third category, Catholic, is composed of couples in which both spouses were members of any Catholic denomination, such as Roman Catholics, Old Catholics, and Free Catholics. The fourth category, “mixed,” comprises couples where one spouse was Catholic and the other liberal or orthodox Protestant. The last category, “other,” contains couples who were Jewish, who belonged to a liberal secessionist denomination, for whom no religious affiliation was specified, or who had no religion (Bras et al. 2010b; Bras 2014). Because orthodox Protestant and Catholic denominations were more stringent in following doctrine and were generally more likely to reject modern forms of birth control, we presume birth intervals for these groups to have been shorter compared with more liberal or moderate denominations (Van Bavel and Kok 2005).

The social class of the household is based on the occupation of the husband as registered in the marriage certificate. If the occupation was missing, it was taken from the population registers. These occupations are coded using the HISCO classification system, a catalogue of historical occupations that corresponds to the International Standard Classification of Occupations. The HISCO codes are then categorized according to the HISCLASS scheme, following Van Leeuwen et al. (2004), into the following categories: (1) higher managers and professionals, (2) lower managers and professionals, including clerks and salesman, (3) foremen and skilled laborers, (4) farmers and fisherman, (5) semi-skilled laborers, (6) unskilled laborers and farm laborers, and (7) unknown occupation. Previous research has shown that the middle and upper classes were the first to postpone childbirths, whereas birth intervals among farmers decreased between 1890 and 1920. Over time, however, the length of birth intervals converged among all social classes (Bras 2014).

### Methods

We use an event history approach to examine whether the lengths of women’s closed birth intervals were associated with the presence of kin and non-kin in their households (Cleves et al. 2010). Event history analysis, also known as survival or duration analysis, models the effects of covariates on the time until the occurrence of a particular event. The chance of the event occurring in the next period is expressed as a coefficient that is dependent on the shape and height of the baseline hazard function. Since the composition of the household continuously changes as people move in and out, our kin covariates are time-varying. The ability of survival analysis to accommodate this type of data makes it a very useful technique. We focus on the effects of kin on the length of birth intervals and model the effects of kin on the transition from the first living child to the next birth, from the second living child to the next birth, and so on. We fit Cox proportional hazard models to examine the effect of the presence of different types of household members on parity progression risk. The Cox proportional hazard models take the following form;

$$ h\left(t\Big|{x}_j\right)={h}_0(t). \exp \left({\upbeta}_x{X}_j\right) $$

where *h*(*t*|*x*_*j*_) denotes the hazard rate, or the chance of having a next birth, in period *t* for the specified vector of time-varying covariates, *h*_0_(*t*) is a non-negative and unspecified baseline hazard function that varies arbitrarily over time and is not dependent on the covariates *X*_*j*_ *,* and β_*x*_ is a vector of unknown regression coefficients to be estimated from the data using maximum likelihood (Cleves et al. 2010). Given that we consider the effect of kin on time until next birth using a sample of closed birth intervals, the cumulative hazard rate increases over time until it is equal to one and all women have given birth. The Cox proportional hazards model allows for estimating the relative hazard rate of women in different groups—for example, living with or without particular types of kin. Both groups have the same baseline hazard at time *t*, but the magnitude of the hazard is multiplied by the exponentiated regression coefficient of each group. Time is measured in months, and a coefficient larger than zero denotes a higher chance of giving birth in period *t*, or in other words, a shorter birth interval.

The Cox model assumes that the estimated hazard ratios are proportional to each other. The assumption of proportionality was tested by examining the Schoenfeld residuals and the proportionality of the log-log plot of the survival rate against the log of time for each variable. These pre-analysis tests indicated that the baseline hazard should be allowed to vary between religions for all models, which ensures that we can still provide reliable estimates for the effects of kin on the hazard rate of childbirth in the period *t*, although the effect of religion is left unspecified. For parity six and up, we estimate one model for all births after the fifth birth and control for net parity, which is the total number of living children at time *t*. In order to ensure proportionality of hazards in this model, we included additional time-varying effects for marriage duration and the death of the previous infant if they survived until they were at least 8 months of age.

Another important assumption of the Cox model is that the risk of parity progression in the sample is randomly distributed across observations. However, since birth intervals are by their nature clustered on the level of the couple, it is likely that parity progression risks are not completely random and the hazard ratio may thus be conditional on the individual frailty of each couple (Cleves et al. 2010). When specifying multiple parities or when a couple has experienced the loss of a previous child, and thus would be observed two or more times in the analysis of a particular parity progression rate, this may be a cause for concern. Testing for the significance of the estimated frailty variance revealed that it was not necessary to include an individual frailty component for each couple. Nevertheless, a robust estimation of variance is recommended given the clustered nature of our observations (Lin and Wei 1989). Goodness of fit was evaluated by examining the Cox-Snell residuals against the Nelson-Aalen cumulative hazard function (Cleves et al. 2010; Cox and Snell 1968). Our analysis is robust for variations in sample size across both geographical areas and time.

## Results

Table 3 provides parameter estimates for the multivariate Cox proportional hazards model by parity. Column 1 contains the results for the time until the birth of a next child, since the birth of the previous child, for all couples who had one living child. Column 2 provides estimates for the length of the birth interval for all couples who had two living children, and so on. The births of children after the fifth living child (parity six and up) are grouped in one Cox proportional hazards model in which the number of living children is controlled for. All models include control variables for unreported household and community-level characteristics.

**Table 3:** Estimated coefficients for the effects of kin presence in the household on the likelihood of second or later-order marital births by parity

Variables	Parity				
	2	3	4	5	6 and up
Household characteristics					
Kin variables^a^					
Wife, both parents	−0.077	−0.089	−0.082	0.153	0.140
Wife, only father	−0.765***	−0.784***	−0.475**	−0.949***	−0.292
Wife, only mother	−0.141	−0.123	−0.012	−0.271†	−0.025
Husband, both parents	−0.034	0.093	−0.262*	0.068	0.172
Husband, only father	−0.680***	−0.697***	−0.893***	−0.452†	−0.203
Husband, only mother	−0.165	−0.022	−0.511**	−0.273†	0.106
Wife’s sister(s)	−0.061	0.153†	−0.029	0.115	−0.047
Husband’s sister(s)	0.011	−0.120	0.204†	−0.118	0.136
Wife’s brother(s)	0.182*	0.242**	0.239*	0.087	−0.028
Husband’s brother(s)	0.115†	0.298***	0.549***	0.270*	−0.137
Other female kin present	−0.023	0.192	−0.236†	0.050	0.083
Other male kin	0.014	−0.039	0.008	−0.008	−0.009
Servant(s)	0.099	0.482**	−0.045	0.526†	0.761***
Boarder(s)	−0.399	−0.429*	0.939***	1.945**	—
Individual characteristics					
Marriage duration (in years)	−0.058***	−0.092***	−0.119***	−0.084***	−0.069***
Marriage duration>18 years^b^					0.354**
Previous infant died					
<8 months	0.329*	0.630***	0.243	0.027	0.303*
>8 months	−0.396*	−0.475***	−0.820***	−0.146	−1.737***
>8 months * time^c^					1.818***
Birth cohort wife^d^					
<1860	−0.125	0.010	0.075	0.183	−0.208*
1861–1870	−0.108	0.031	0.050	0.040	−0.235*
1881–1890	−0.262**	−0.132	−0.021	−0.086	−0.365***
1891–1900	−0.423***	−0.239**	−0.238*	−0.243†	−0.429***
>1900	−0.627***	−0.487***	−0.466***	−0.421**	−0.487***
Crude parity	0.021	0.239***	0.246***	0.241***	0.091**
Net parity					0.038
Births (*N*)	2331	1727	1204	867	1923
Couples (*N*)	2331	1727	1204	867	629
Observation periods (*N*)	4048	2918	1904	1286	3414

Cox proportional hazards models, coefficients reported. Cluster robust standard errors, adjusted for dependence among births of the same couple, stratified on religion, Breslow approximation for tied survival times. A positive sign indicates a shorter birth interval for the associated covariate. All models control for occupation, religion, urbanization, community characteristics, age, and spousal age gap (coefficients for those variables are available from authors upon request)

Source: Historical Sample of The Netherlands, release 2007

† *p* < 0.1, * *p* < 0.05, ** *p* < 0.01, *** *p* < 0.001, — not present

^a^Reference: none present

^b^Square root of marriage duration after 18 years

^c^Linear interaction with analysis time after 24 months

^d^Reference: 1871–1880

Our results indicate that for later-order births, from parity six and up, co-residence with kin was not significantly associated with longer or shorter birth intervals. For lower parities, living with both parents of either the husband or the wife did not affect the time until next birth, although living with the husband’s parents had a small delaying effect on the birth of the fourth child. In contrast, living with a widowed father of either spouse significantly reduced parity transition rates. The birth intervals of women living with a widowed father were about twice as long as birth intervals of women who did not live with a widowed father.^2^ Conversely, living with a widowed mother in the household did not significantly affect time until next birth, with the exception of the fourth parity in the case of a husband’s widowed mother.

The length of time between births was at least 20% shorter for women who lived with at least one brother or brother-in-law, compared with women who did not live with a brother or brother-in-law in the same household. The positive effect of the presence of the husband’s brother was particularly high for women experiencing the transition from the third to the fourth child, for whom the parity transition rate was almost twice as large as for other parities. Strikingly, in contrast to living with brothers, living with sisters of either of the spouses did not significantly affect parity transition rates.

Other types of co-resident kin, such as aunts or uncles, had no systematic effect on the duration of the transition to the next birth. The presence of non-kin did affect birth intervals, but their effect was not stable over the reproductive life course. The presence of servants affected only the waiting time until the birth of the third child and the birth intervals of fifth and later-order children. Co-resident boarders and lodgers had a significant delaying effect on the birth interval for the third parity, but shortened the birth interval for the fourth and fifth parity. Keeping in mind the low number of observations of non-kin in our sample, these findings should be interpreted with care.^3^

The time until next birth was longer for couples who were married for a longer period of time, as was expected. However, for women who had at least six children, we observe that parity transition rates increased after 18 years of marriage compared with women who were married for less than 18 years. Crude parity indicates the total number of children that have been born to a woman. As would be expected, women who had experienced a larger number of pregnancies had shorter intervals between births. Similar to findings by Van Bavel and Kok (2004, 2010), parity transition rates of women whose last child died before it was 8 months old were significantly higher than those of women whose last child survived. The death of the previous infant at 8 months of age or later delayed the birth of a next child, although after 24 months the chance of having a next birth was significantly higher. In line with the observations presented in Table 1, birth intervals were longer for women born in later cohorts compared with those born in the reference period 1871–1880.

## Discussion

In human behavior, as in the behavior of other animals, the provision of support to genetically related kin is expected to confer an indirect advantage in terms of inclusive fitness (Hamilton 1964a, b; Hrdy 2008, 2009; Gurven et al. 2001). It follows that couples who can rely on close kin members for support are more likely to raise more or better-quality children than couples who are not surrounded by close kin (Kramer 2010; Salmon and Shackelford 2008). In this study we have investigated whether the presence of co-resident kin and non-kin affected the length of birth intervals for 2628 Dutch women born between 1842 and 1920. Our point of departure was the broad hypothesis that, on the basis of inclusive fitness theory, all close kin members would exert a positive influence on reproductive outcomes by enabling the women in our sample to have shorter intervals between births. The effects of kin on fertility were expected to be positively associated with the strength of the genetic bond between kin, whereas genetically more distant kin would have a minor effect on reproduction. Furthermore, kin influences were hypothesized to be stronger during the first, formative years of the family when alloparental support could compensate for the inexperience among new parents learning about the nutritional needs of newborns or for the extra work needed to feed an additional mouth.

Using continuous-time data on household composition as a proxy for cooperative behavior, we find that co-resident kin had different effects on fertility at different stages of the reproductive life span of women. The effects of kin were not significant for higher parities. This finding may provide a partial explanation for the variations in kin effects on fertility that have been observed in the literature in which only measures of complete fertility outcomes are taken into account (see Sear and Coall 2011 for an extensive overview of the literature). However, without knowledge of the distribution of resources and care among household members over time, it is difficult to infer from our data why the effects of kin were only significant in the early parities. As Lyngstad and Prskawetz (2010) argue in their study of Swedish sibling pairs born in the mid-twentieth century, the decrease of kin influences might be attributable to uncertainties around the process of entering parenthood, but possibly also to changes in both the different roles of kin within the household and their ability to provide the couple with any form of support.

Our findings suggest that co-resident kin did not affect reproductive outcomes of Dutch women in a uniform way. In contrast to other empirical findings, we find that parity progression rates were not significantly affected by the presence of widowed mothers or both parents of either spouse (Hawkes et al. 1998; Hawkes 2003; Pollet et al. 2007; Voland and Beise 2002). The absence of a positive “grandmother effect” regarding the length of birth intervals is also observed in other studies (e.g., Hill and Hurtado 2009). However, Dutch women who lived with their widowed father or the widowed father of their husband experienced significantly longer birth intervals than women living without a widowed father. Whereas some studies have shown no effect of fathers on fitness outcomes (Borgerhoff Mulder 2007; Sear and Coall 2011), in others a negative effect of fathers on their daughters’ reproductive behavior has been observed, in particular in relation to offspring survival chances (Kemkes-Grottenthaler 2005). Our findings concerning the delaying effect of co-resident widowed fathers on parity progression provide support for the confidence of paternity hypothesis, which suggests that uncertainty over genetic relatedness will lower the extent of cooperative behavior to offspring (Strassmann and Garrard 2011). However, on its own this hypothesis has received little empirical support in explaining differences in the influence of parents on demographic outcomes (Euler and Weitzel 1996; Pashos and McBurney 2008). The delaying effect of widowed fathers might also be attributable to the notion that fathers consumed a relatively large share of the couples’ resources, especially care, for themselves while providing the couple with little support or few pronatal incentives (see also Kemkes-Grottenthaler 2005). Recent empirical studies show that kin effects are indeed modified by conflicts over resources (Borgerhoff Mulder 2007; Schaffnit and Sear 2014). With regard to siblings, our findings indicate that the presence of brothers, but not the presence of sisters, was positively associated with parity progression. This observation suggests that the additional resources that brothers brought into the household had enabling effects on couples’ reproductive outcomes (Becker 1981; Becker and Barro 1988; Feng et al. 2010).

Our study contributes to the growing literature on empirical approaches to evolutionary theories of demographic behavior. The findings presented here raise further questions concerning the role and position of kin members within the household as well as the extent of their cooperative behavior, such as provisioning of care or contributions to household income, which is difficult to infer from kin presence alone (see, e.g., Schaffnit and Sear 2014). In addition, owing to the nature of the HSN data, our observations are limited to household members, but kin living outside the household may also have affected the women’s reproductive careers (Johow and Voland 2012). These issues further complicate the connection between the assumptions on which our hypotheses are based and our findings. Although people may receive fitness benefits from higher reproductive outcomes of their kin, shorter birth intervals are not by definition in the woman’s interest and in fact may lower the quality of offspring (Borgerhoff Mulder 2000; Conde-Agudelo et al. 2006, 2007; Palloni and Millman 1986). Nonetheless, our findings do clearly indicate that the presence of widowed fathers and brothers affected parity progression rates, leading to the conclusion that reproductive outcomes were subject to the distribution of resources and care within the household.

Inclusive fitness theory enables us to understand the motives underlying the behavior of household members toward genetically related others, but actual demographic outcomes are determined by the specific historical, social, economic, and spatial conditions of the household, as well as maternal health and the extent of cooperative behavior of kin that enables women to give birth. Future research on the interaction between wealth and kin influence, or differences in cultural norms concerning kinship and reproduction, which lie beyond the scope of this study, may further illuminate variations in the influence of kin on reproductive outcomes.

## Footnotes

1 See also Janssens (1993:64), who estimates that around 0.2 % of all live births are not listed in the population registers. Many of these children passed away very soon after birth. Although their omission produces an upward bias in the average length of birth intervals, it is unlikely to greatly affect our conclusions given the relatively small number of missing observations.

2 The exponentiated coefficients represent the chance, or hazard, of a birth occurring in period *t* relative to the reference category. Here *e* ^− 0.7^ implies a hazard rate of 0.5, or in other words, a doubling of the length of the birth interval.

3 Our findings concerning the influence of kin are the same when non-kin are excluded from our analyses.
